# Impact of Reactant Dissolution in the Kinetics of a Catalytic Hydrogenation for the Production of Argatroban

**DOI:** 10.1021/acs.oprd.4c00479

**Published:** 2025-03-12

**Authors:** Filippo Nanto, Dario Ciato, Mariano Stivanello, Paolo Canu

**Affiliations:** † Industrial Engineering Department, University of Padova, Via Marzolo 9, Padova 35131, Italy; ‡ Lundbeck Pharmaceuticals Italy, Quarta Strada 2, Padova 35129, Italy

**Keywords:** catalytic hydrogenation, reagent dissolution, mass transfer and kinetics, quantitative analysis, argatroban

## Abstract

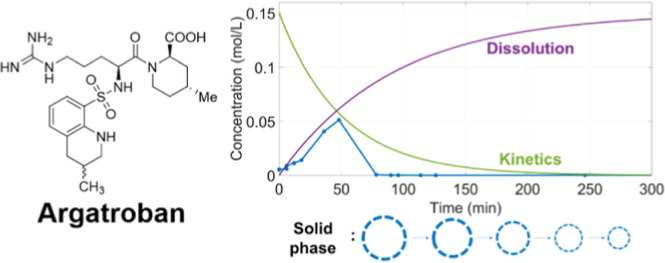

An experimental study was performed for a fed-batch catalytic hydrogenation for the production of Argatroban. The penultimate expensive and scarcely available intermediate is characterized by a slow dissolution rate that evolves in parallel with the reaction process. The study investigated the coupling between the reaction and dissolution kinetics. In these circumstances, the standard Area Percentage method in HPLC was found to be misleading, requiring calibration and then absolute peak area measurements to correctly identify the dissolution rate and thus the actual chemical kinetics. Experiments quantified the role of the temperature, stirring rate, and catalyst loading. Shifting from 40 to 80 °C reduced the batch time by 58%, although higher temperatures promoted the formation of undesired impurities. Stirring rate controlled the initial reaction phases when reagent dissolution is critical. Catalyst loading is key in reducing batch time. The increase in catalyst loading was proved to affect the reagent dissolution rate, by increasing the collision frequency between reagent and catalyst particles. A refined first-principles model, incorporating the effect of the catalyst amount on the dissolution mass transfer coefficient, significantly improved the accuracy of dissolution predictions and enabled better identification of the intrinsic reaction kinetics. The addition of a microkinetic description further improved the predictions of intermediates and products.

## 1 Introduction

Processes in the pharmaceutical industry are often carried out batchwise, and frequently solids are involved. The presence of solids is one of the most common features contributing to the rising process inefficiency. It is also one of the main factors limiting the shift from batch to continuous operations.^1,2^ Solid phases are overwhelmingly present during the isolation phases of an API, in steps such as drying, filtration, and centrifuging. However, solids also impact the API synthesis. Indeed, solids can be found in the reaction section, as catalysts, reagents, or products, and they could undergo dissolution, precipitation, and crystallization, in parallel with the reaction. In these cases, the presence of a dispersed solid phase in a liquid creates additional difficulties in understanding, scaling-up, and managing the process at an industrial scale.^3,4^ Difficulties are associated with mixing, fluid mechanics, mass and heat transfer rates, suspension and phase continuity, and structural changes of the solid material over the time.

The body of the literature addressing coupled reactions and solid dissolution within the pharmaceutical industry is scarce. Grénman et al. studied the homogeneous reaction between a sparingly soluble solid compound, 1,2,4-triazole as a sodium salt, and a complex substituted aliphatic halide, in a 1 L batch reactor.^5^ The effect of the temperature and 1,2,4-triazole concentration on conversion was investigated. Within the same time span, an increase of 50 °C increased the conversion from 70 to 100%. Increasing the excess of the initial solid did not affect the reaction rate and selectivity. Indeed, an excess of solids saturated the reaction mixture over the whole batch, avoiding the need to account for total reagent consumption, i.e., predict the end of dissolution. This cannot be the case with expensive, scarcely available reactants that cannot be used in excess, as in our case. Since the reaction was not catalyzed and the amount of the solid reagent is above its solubility, mass transfer in the liquid was not an issue. Sano et al. analyzed the production of a pharmaceutical intermediate in a reaction calorimeter, with one reagent gradually dissolving in methanol.^6^ The other reagent, aqueous dimethylamine, was slowly added. Again, the reaction was not based on a solid catalyst and took place in the solvent phase. The authors identified that during the early period of the reaction, the dissolution of the reagent was the rate-determining step (RDS). For this reason, the effect on dissolution of the stirring rate (100–300 rpm) and dimethylamine addition time (1–3 h) was studied. In both cases, the dissolution time of the reagent did not change. They concluded that dissolution was affected only by the reaction progress. Unfortunately, these and other studies involve the coupling of reaction with dissolution–crystallization, in two-phase reactions.^7,8^

**Figure 1:**
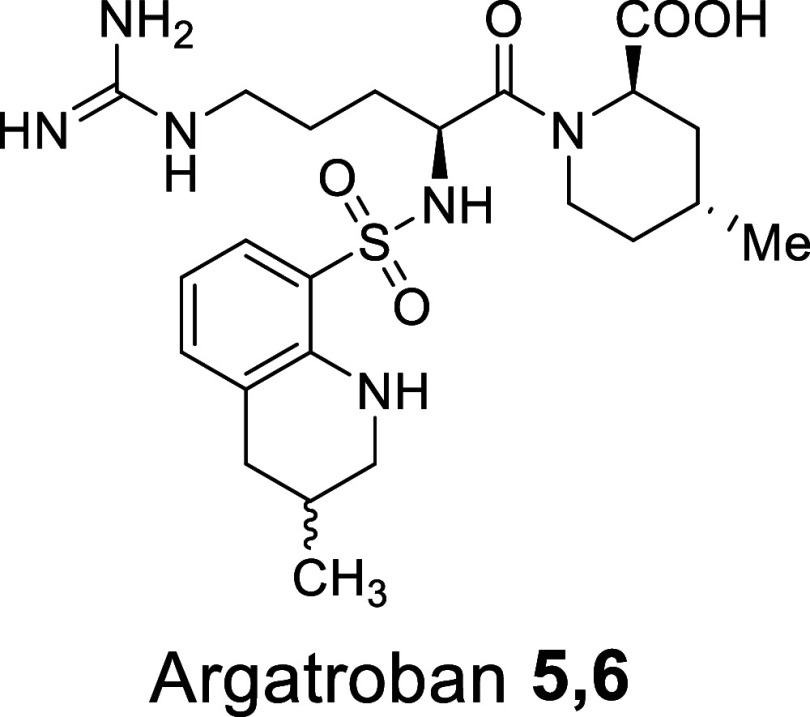
Structure of Argatroban.

In this work, a dissolving reagent impacts a more complex process, a catalytic two-step hydrogenation that involves four phases: a liquid (the solvent), a gas (H_2_), and two solids (the reagent and the catalyst). The process under analysis is the final synthetic step in the production of Argatroban **5,6** (Figure 1). Argatroban, (2*R*,4*R*)-4-methyl-1-[N2-(3-methyl-1,2,3,4-tetrahydro-8-quinolinesulfonyl)-*l*-arginyl]-2-piperidinecarboxylic acid, is a highly selective direct thrombin inhibitor. It is commercially available as a mixture of stereoisomers in a ratio of 64/36 of **5** to **6**. It is an anticoagulant used for the treatment and prophylaxis of thrombosis in patients with heparin-induced thrombocytopenia (HIT). Compared with **5,** isomer **6** significantly prolongs coagulation time of whole blood (CT), recalcification time, kaolin partial thromboplastin time (APTT), pro-time prothrombin time, and thrombin time and reduces the platelet adhesion rate and platelet aggregation rate. This stronger anticoagulant effect allows for a lower therapeutic dose, making compound **6** suitable for treating or preventing thrombosis and inhibiting platelet aggregation. It was discovered and developed by Mitsubishi and it is now a generic API.^9−11^

**Scheme 1:**
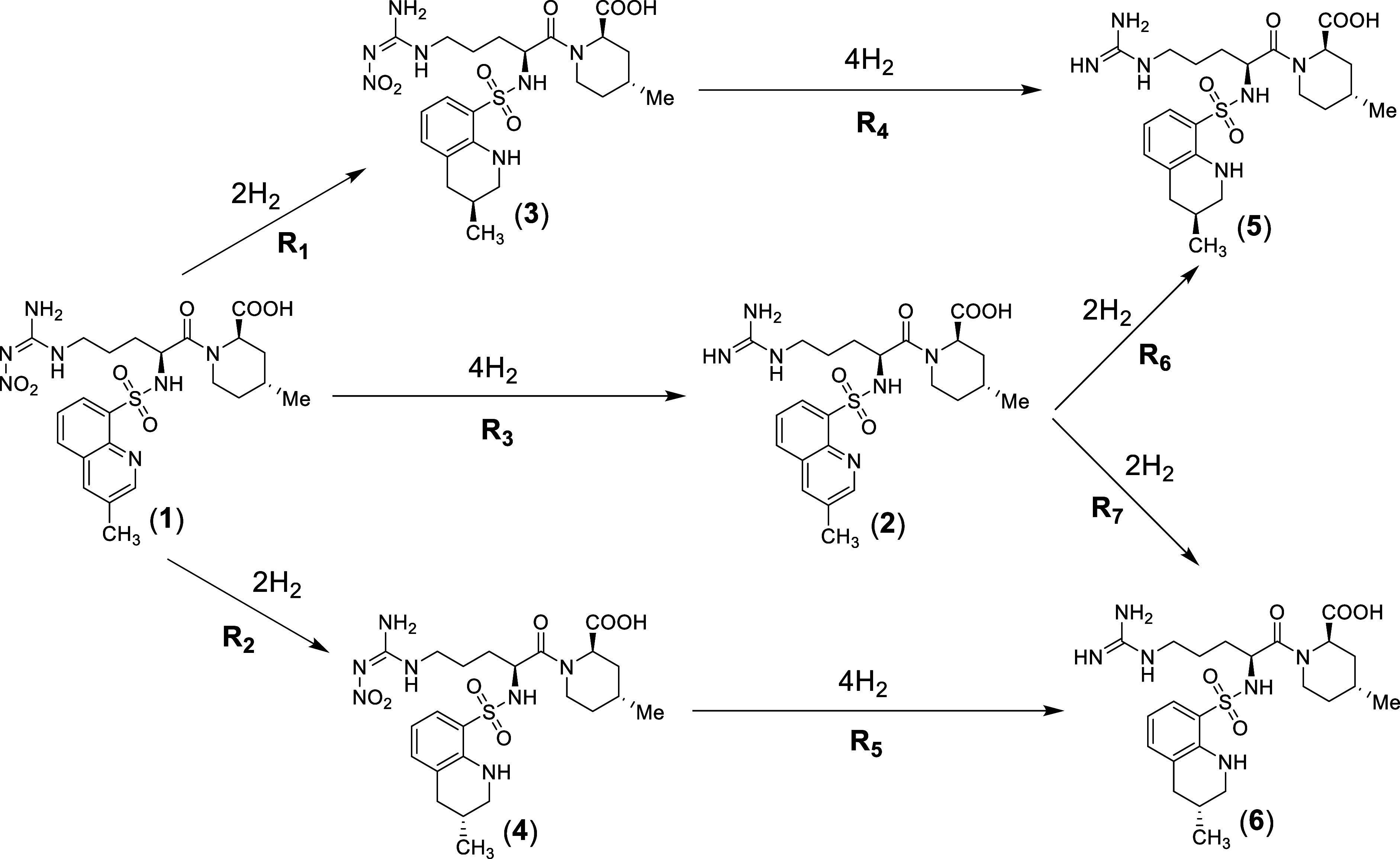
Kinetic Pathway to Obtain a Mixture of **5** and **6** from **1**, through the Intermediates **2**, **3**, and **4**

The starting material **1** is a costly and scarcely available intermediate since it is obtained through a complex multistep synthetic route (Scheme 1). The investigation of the kinetics and scale-up was severely constrained by the raw material availability, which is a common paradigm of the R&D routine activity in the pharma industry. The importance of the correct quantitative analysis for these processes was first assessed. Then, the effect of temperature, stirring rate, and catalyst mass on product purity and process time was analyzed at a laboratory scale. The average distance between particles of the two solid phases present in the reaction mixture was estimated, to check if collisions between particles could play a role in improving the dissolution of the reagent. Additionally, a previously developed first-principle model, that demonstrated predictive capabilities across a range of operating conditions, such as variations in temperature, stirring rate, reactor scale, and geometry,^12^ was refined to account for the improvement of dissolution resulting from the presence of catalyst particles. A correlation between the solid–liquid mass transfer coefficient for dissolution and the catalyst amount was added to improve the model’s accuracy, together with a microkinetic description of all reaction steps. The developed model can be used to inform and organize production campaigns for the utilization of multipurpose reactions and to perform in-silico process optimization for pilot and production reactors, to find optimal operating points and to simplify process procedures.^12^

## 2 Experimental Section

### 2.1 Synthetic Route

The reaction studied is a sequential reduction by H_2_ of a nitro-guanidine group and a quinolinic ring of a key intermediate, **1**, and it is the final step in the synthesis of Argatroban **5,6**. The final product is obtained as a mixture of two stereoisomers (**5** and **6**) in a defined ratio of 64/36. The formation of a fourth chiral center during hydrogenation of the quinolinic ring produces these isomers. Notably, the ratio between the two isomers remains constant irrespective of variations in the process conditions, indicating thermodynamic control over their distribution. The simplified kinetic pathway is outlined in Scheme 1.

The two hydrogenation steps can occur in two alternative and competitive sequences, giving rise to a mixed parallel-series mechanism. Three intermediates can be generated, **3/4** and **2**, from the reduction of **1** with H_2_. **3/4** is a mixture of two stereoisomers, generated by the partial hydrogenation of the quinolinic ring, while **2** is a single compound generated by reduction and cleavage of the nitro group in the guanidine moiety. The solvent is a mixture of methanol, acetic acid, and water (0.78:0.19:0.03). It was selected for a rapid solubilization of H_2_ in the liquid, so that it is not limiting for the reaction.^12^ For a detailed discussion about H_2_ uptake in this solvent system, see Section 4.2. The catalyst in use for the process is Pd/C, which is the established catalyst for the synthesis of Argatroban, as it preserves the selectivity toward the reduction of the quinolinic ring.^9,11,13^ A 5% palladium loading was selected, as it was found to provide the optimal balance between purity (exceeding 97%) and overall yield, compared to a 10% Pd loading. Reducing the Pd concentration to below 5% would result in a significantly slower process, making it impractical for industrial production.

### 2.2 Reactor

A 500 mL jacketed glass autoclave (Büchi) was used. It has a cylindrical shape and a round bottom. This vessel was selected due to its geometrical similarity to the industrial reactor currently used for commercial-scale synthesis. Due to the small volume, hydrogen was fed directly into the autoclave headspace and then dissolved into the liquid both through the vortex generated by the 6-blade disk turbine (Rushton) and by a hollow shaft, to redisperse H_2_ from the gas above the liquid mixture. Traditional baffles are absent; the limited diameter allows only a temperature probe and a pipe for sampling, which help avoid the liquid mixture rotating as a solid body. Further information about both reactors’ geometry is reported in Figure S1 and Table S1 in the Supporting Information.

### 2.3 Procedures

The empty vessel was rinsed with a 5% aqueous solution of nitric acid to remove particles of the catalyst from previous tests and then with distilled water to achieve neutrality before the beginning of each experiment. Nitrogen/vacuum cycles were performed with the empty vessel to remove O_2_, which could deactivate the catalyst. Subsequently, the vessel was brought to atmospheric pressure by using nitrogen. Afterward, solid **1** and catalyst were loaded and additional nitrogen/vacuum cycles were performed. Previously degassed solvents were then poured into the vessel. At this stage, the obtained suspension was gently stirred, cooled at 10 °C, and H_2_/vacuum cycle was performed to remove residual nitrogen. After all these steps, the process begins; temperature was raised at 40 °C, and the stirring rate increased at 300 rpm. After 40 min under these conditions, both process temperature and stirring rate were increased (40 → 80 °C, 300 → 600 rpm), the first within 40 min, and the second in about 5 min. These stirring speeds were determined using a downscaling rule based on achieving fully turbulent conditions for the entire duration of the process. In the already validated industrial process, the stirring speed varies between 125 and 250 rpm in the production reactor, ensuring fully turbulent conditions at all times. At the laboratory scale, the minimum stirring speed required to achieve a fully turbulent flow is approximately 300 rpm. To remain consistent with the industrial procedure, the stirring speed was doubled to 600 rpm on the same ramp.

In addition to experiments of synthesis of **5** and **6**, the quantitative evaluation of the reactant **1** dissolution rate and extent (solubility) in the solvent, without reaction (no catalyst), was measured at different temperatures in dedicated experiments. For that purpose, the glass jacketed autoclave was rinsed three times with methanol, then fluxed with N_2_, and vacuumed, for 15 min each, to remove the residual solvent. 10 g of **1** and 100 mL of the solvent were charged. A large excess of **1** was used to ensure saturation. Stirring at 400 rpm, the suspension was rapidly heated to the set temperature. Liquid concentration analysis was always carried out via HPLC.

Measurements of H_2_ solubility (rate and extent) in the solvent mixture used were also carried out at the laboratory scale, at different temperatures, and with a stirring rate of 600 rpm. The pressure decay in the gas reservoir connected to the reactor headspace was monitored by a pressure sensor. The reactor was pressurized without stirring, assuming negligible H_2_ absorption into the stagnant liquid. When the stirrer is activated, dissolution begins and the headspace pressure decreases, until stabilization, once the saturation of liquid with H_2_ is achieved.

While steady-state values provide the solubility of **1** and H_2_ in the liquid, the rates of dissolution of **1** and H_2_ are determined by the time evolution, up to equilibrium, through the appropriate mass balances in the liquid (for **1**) and in the gas (for H_2_), applied to these nonreactive tests. Details are reported elsewhere.^12^

It is essential to highlight that H_2_ is not fed with a constant flow; the flow of H_2_ is regulated by pressure controllers, when needed, to keep the set pressure. The values of all process parameters are reported in Table S2 in the Supporting Information.

### 2.4 Materials

All materials used for the experimental campaign were approved according to the internal specifications of the quality department. Starting reagent **1** is internally manufactured. 5% Pd/C catalyst was provided by Faggi Enrico SpA (approximately 110 m^2^/g_Pd_, 94 μm mean diameter). Methanol and glacial acetic acid were purchased from MetMed SrL and proFagus GmbH. Over 99.9% purity N_2_ and H_2_ were supplied by SIAD SpA.

### 2.5 Process Data Measurement

A temperature probe, that ranges from 0 to 200 °C with ±1% full-scale precision, was inserted inside the liquid mixture, and the stirring rate could be varied from 0 to 1400 rpm with ±5% full-scale precision.

To evaluate the concentration of species in the solvent mixture, UV-HPLC (Waters e2695) was used because molecules are characterized by chromophore groups. A detailed description of the HPLC method utilized is provided in Section S1.3 in the Supporting Information. With this analytical method, it is common practice to report measurements following the liquid chromatography area percentage (LCAP) method, in particular with many species, possibly not fully identified.^14,15^ It allows skipping calibration steps, but it could lead to erroneous assumption of the response factors (area/amount) equal for all species, which may be particularly false for hydrogenation products. This approach is convenient and swift when evaluating the product fraction, which often needs to be in a defined ratio, but it does not give an indication of the absolute quantity of single species. Indeed, if mass transfer between different phases occurs, that selectively adds and removes species from the liquid, using LCAP important information about the process may be neglected or lost. Accordingly, LCAP has been critically compared with the absolute peak area method, as discussed in the Results and Discussion section, using calibrations.

## 3 Kinetic and Mass Transfer Model

The reaction system considered involves four distinct phases that are characterized by selective mass transfer between them. A comprehensive mechanistic first-principles model was developed to elucidate the interplay between the mass transfer phenomena, notably the dissolution of **1**, and the complex kinetics pathway governing the hydrogenation reaction.^12^ The model incorporates all pertinent mass transfer rates between phases. It accounts for the influence of temperature, stirring rate, reactor dimensions, and impeller type on the hydrogenation process. Its development and validation are thoroughly described in another work. In the current study, the model is expanded to include the influence of catalyst loading, and different kinetic models are also considered to improve the explanation of the experimental data and its predictive capability.

### 3.1 Catalyst Amount Effect

The mass balances for all of the species present in the liquid phase follow the expression
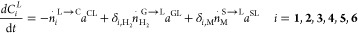
1where *C*_*i*_^L^ is the concentration of a single species in the liquid mixture and  is the molar flux of a single species from the generic phase α to generic phase β (G = gas, L = liquid, C = catalyst, S = solid reagent). Since the mass transfer from the gas and the solid reactant applies only to either H_2_ or **1**, Kronecker’s delta (δ_*ij*,_ equal to 1 for *i* = *j* and 0 for *i ≠ j*) is used in eq 1 for convenience, as it allows the system of equations to be expressed as a single unified equation. The liquid phase is assumed to preserve its volume along the reaction progress. Each specific interface is based on the liquid volume, i.e., *a*^αL^ = *A*^αL^/*V*^L^. Specifically, the interfacial area between the catalyst and the liquid can be formulated as
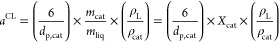
2*a*^CL^ is directly proportional to the catalyst loading *X*_cat_, defined as the ratio of the catalyst mass to the solvent mass, and inversely proportional to the diameter of the Pd/C particles. Increasing the catalyst loading is expected to extend the surface available for reaction linearly if the catalyst particle size does not change.

When H_2_ saturates the liquid, the reactants are completely dissolved at the reaction onset, and also products remain dissolved, as typical for most hydrogenation reactions, increasing the catalyst loading results in a proportional increase in the rate of species evolution (*d*C_*i*_^L^/d*t*). When this simple rule was implemented in the model and the predictions were compared with experimental data for different catalyst loadings, the model predictions did not reasonably explain the experimental results. Departures were more relevant for dissolving reagent **1**, subsequently propagating to intermediates and final products.

The coexistence of two distinct solid phases, **1** and the catalyst, may explain why the amount of catalyst does not impact linearly on the rate of reaction. The presence of catalyst particles could affect the dissolution of reagent **1**; a higher concentration of solids in the solvent causes more frequent collisions between particles, improving the rate of transfer of the reagent from the solid to the liquid phase, especially when the two solid phases exhibit significant differences in particle size. The movement of smaller catalyst particles near the reagent particles induces a convective transport that speeds up liquid renewal near the solid reagent, improving the mass transfer rate compared to purely diffusive transport at the interface.

To check whether collisions between particles have to be accounted for, the average distance between particles has been estimated for both solid **1** and the catalyst. The number of particles of each phase can be calculated considering the total mass of solid charged, divided by the mass of a single particle, which is calculated using its mean diameter.
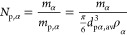
3

Assuming the particles are uniformly distributed in the solvent, the average distance between particles (Δ), accounting for their own steric encumbrance, can then be estimated as
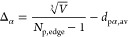
4where *V* is the unit reaction volume containing *N*_p_ particles. If the average distance between the particles is at least comparable to the particle diameter, it is reasonable to assume that collisions between particles of **1** and the catalyst, and collisions between **1** particles as well, could occur frequently. These collisions certainly increase the mass transfer coefficient for dissolution (*k*_SL_) as they can break larger particles or conglomerates, creating additional surface and accelerating its transfer to the liquid phase (the mass transfer coefficient increases with decreasing particle diameter).

To account for these fluid dynamic and physical effects, the following correlation was included in the model, which connects the volumetric mass transfer coefficient for the dissolution of **1** to the amount of the catalyst present in the liquid mixture. β_1_ and β_2_ are positive parameters that have to be tuned on experimental data.
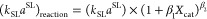
5eq 5 is a semiempirical correlation that accounts for the additional fluid dynamic phenomena influencing reagent dissolution, as discussed in this section. These phenomena are typically excluded from conventional analyses of solid dissolution, which are predominantly studied as standalone processes. It provides a correlation between *k*_SL_a^SL^ and the catalyst loading (*X*_cat_) without accounting for reactor configuration or impeller geometry. However, the model incorporates the influence of the reactor diameter, volume, and impeller type through the Zwietering coefficient (*S*) in the calculation of the mass transfer coefficient, enabling consideration of different impellers or reactor configurations.^12^

### 3.2 Kinetic Models

The kinetics is summarized by the species production rates, *r*_*i*_, in the material balances, determined by the combinations of all of the reactions where each species is involved. Specifically
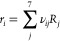
6where *R*_*j*_ are the rates of the catalytic reactions. In the previously developed model,^12^*R*_*j*_ were evaluated as global reactions, i.e., the actual molecular mechanisms were not described in detail, with the following expressions
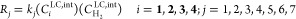
7

To improve the model accuracy and predictive capability under a wider range of process conditions and variation, a microkinetic Langmuir–Hinshelwood–Hougen–Watson (LHHW)-type model was also considered. The synthetic pathway for the production of **5** and **6** consists of two main reactions, the hydrogenation of a –NO_2_ group and the hydrogenation of a quinolinic ring.

Regarding the reduction of the –NO_2_ group, a simplified expression based on literature models developed for the hydrogenation of nitrobenzene on palladium was used.^16^
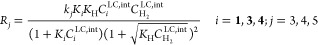
8

The main assumptions for the formulation of this expression are of noncompetitive adsorption between hydrogen and organic species, dissociative adsorption for hydrogen, partial equilibrium for adsorption and desorption, as well as for all elementary reaction steps except for the RDS, which is the reaction between an adsorbed hydrogen atom and the adsorbed nitronic acid intermediate formed. These assumptions are common for most of organic compound’s hydrogenations in the liquid phase.^17^ The equilibrium constant that should be present in the numerator of eq 8 has been included in the kinetic constant.

The same assumptions and simplifications were used to develop a model for the hydrogenation of the quinolinic ring, wherein the RDS was identified as the reduction of the first double bond in the ring, as the intermediate substrate with hydrogenation of only one of the double bonds in the quinolinic ring was not detected. This assumption aligns with other studies on sequential hydrogenations of double bonds over Pd/C catalysts.^17,18^
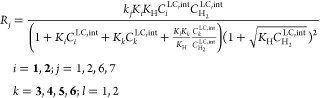
9

A detailed description of the derivation of this expression is provided in the Supporting Information.

Adsorption constants (*K*_i_) have been calculated from the heat of chemisorption, *Q*, by using the following expression
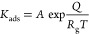
10

Since the rate of H_2_ dissolution in the liquid was found to be much faster that its rate of consumption, the H_2_ concentration at the surface of the catalyst, , is expected to approach the H_2_ solubility, ; note that the temperature affects the solubility, and it is explicitly involved in the rate laws.

The overall model, despite its substantial simplifications and assumptions, requires the estimation of 34 parameters to account for temperature variations during the experiments. While more complex and comprehensive formulations were also developed for each reaction rate, the elevated number of parameters would likely lead to data overfitting. These extended expressions are provided in Section S1.6 in the Supporting Information.

Rates of reactions contribute to each species according to the stoichiometric matrix
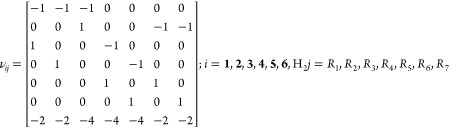
11

## 4 Results and Discussion

### 4.1 Quantification of Liquid Mixture Composition

**Figure 2:**
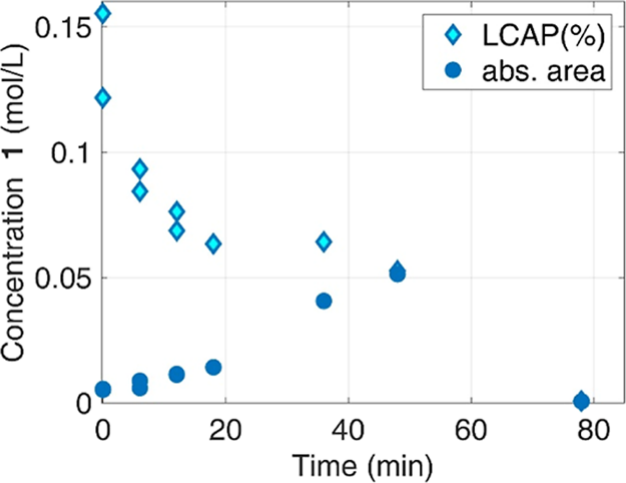
Concentration of **1** over time in the reference test. LCAP vs absolute peak area.

As anticipated, when reactive species (such as H_2_ and **1**) also appear in phases other than the reaction environment (the solvent), the analytical method must capture the actual concentrations in the liquid and to quantify the fluxes between all phases. Two methods of HPLC data analysis are compared: the frequently used LCAP (relative peak areas) and the absolute peak area, coupled with calibration. The relative area method is based on the assumption of comparable retention by the separation column of all species, which is indeed reasonable, for similar molecules; unfortunately, the method provides the relative, not the absolute amount of species in the mixture, overlooking additions (or losses) from (or to) other phases. Figure 2 highlights the substantial difference between the two methods in the evaluation of the concentration of **1** in the liquid during a reference test.

If LCAP is used, then the dissolution (i.e., the progressive increase of **1** concentration in the liquid) is not apparent. The concentration trend reported with LCAP suggests a reactant consumption since the beginning, while the correct evolution of **1** in the solvent is the result of an increase, because of dissolution progress, and consumption, because of the reaction. In addition, the reactant consumption reported by LCAP analysis shows an evident change in the consumption rate, at about 20 min; it could suggest a change in the reaction mechanism, or in the kinetics. In conclusion, the analysis of chromatography data by relative areas, just because it is simpler and avoids difficult or impossible calibrations, is not simply a lack of precision but leads to a dramatic misunderstanding of the physical and chemical processes that actually take place in the reactor. The use of an internal standard, while improving the precision of measuring the total number of moles in the liquid phase, does not resolve these challenges. Specifically, employing an internal standard in combination with an LCAP-type approach would neither ensure the closure of the mass balance nor accurately determine the distribution of the reagent between the liquid and solid phases. This limitation arises because, for a significant portion of the process, reagent **1** is present in both phases. Consequently, a calibration curve is required to accurately quantify the amount of the reagent present in the liquid phase at any given time.

Precisely for this reason, all of the collected data in this work have been analyzed through the absolute peak area, coupled with calibration lines. It is recommended that this check should always be carried out in an industrial environment at the beginning of each experimental campaign; partial dissolution or secondary-phase formation and/or depletion (e.g., crystallization) may be easily overlooked.

A mass conservation check is another useful method to spot the unnoticed coexistence of multiple phases. The measured total moles needed to be equal to what is to be expected from the reaction stoichiometry. In this case, the reaction mechanism outlined in Scheme 1 shows that **1** is hydrogenated in several positions, but its core structure is preserved in all species, both intermediates and products (**2, 3, 4, 5,** and **6**). That is a stoichiometric constraint. Accordingly, the sum of all of the moles of organic species, in all phases, at any time must be equal to the initial amount of **1** fed. After complete dissolution, the total amount of moles present in the liquid must match the initial **1** feed, while departures are expected during its dissolution. The total number of moles in the liquid was calculated for each sample along the reaction course. In the beginning, the moles in the liquid are approximately zero, because only **1** is present, in solid form. Then, it incrementally reaches the total amount initially charged as solid **1**, meaning that the solid is dissolving and transforming into intermediates and products. Afterward, it is to be expected that the total moles of organic species in the experimental measurements equal the initial value, within the experimental uncertainty. Systematic deviations beyond the irreducible experimental error must be corrected to match the stoichiometric constraint. The relative error between the theoretical and measured total moles in the liquid leads to a correction factor
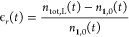
12that allows one to rescale each measurement to be consistent with the stoichiometry as
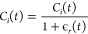
13

The correction was applied to each experiment. The limitation of this correction is that it is based on liquid-phase concentrations alone; it cannot be applied at the beginning of the process, before dissolution of **1** is complete, when some **1** is still solid and impossible to be quantified online. A set of raw experimental data is shown in Figure S4 in the Supporting Information.

### 4.2 Liquid Solubility and Dissolution

**Figure 3:**
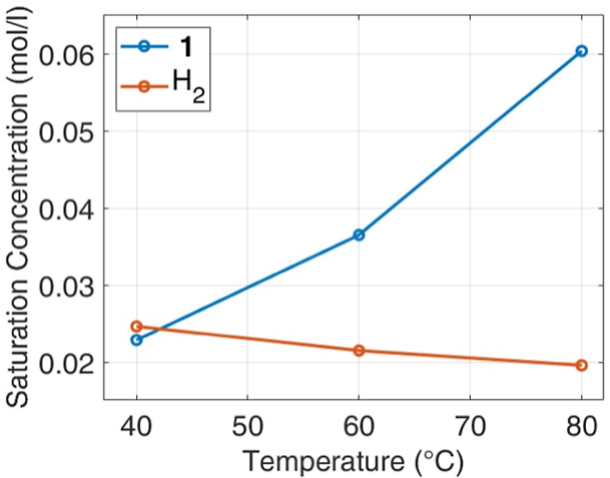
H_2_ and **1** saturation concentration, at different temperatures. *P* = 8.5 bar.

Two species, **1** and H_2_, are present in a different phase other than the liquid, and both have a limited solubility in the selected solvent mixture. Dedicated experiments have been carried out to separately evaluate physical properties and processes such as solubility and dissolution rate, with the experimental procedures and measurements described in the Procedures Section. Results are shown in Figure 3; both saturation concentrations are in the same order of magnitude at 40 °C; but the temperature effect is opposite, slightly decreasing the H_2_ solubility and increasing the **1** one; at 80 °C, the **1** solubility is three times the one of H_2_.

H_2_ concentration trend is opposite to the expected solubility in pure alcohols.^19^ However, some water is also present in the solvent mixture, which is adsorbed onto the catalyst. Its presence, even in small amounts, may impact H_2_ solubility. Given the comparable solubility of both **1** and H_2_, the time required to reach saturation could be significant in understanding the process controlling regime.

**Figure 4:**
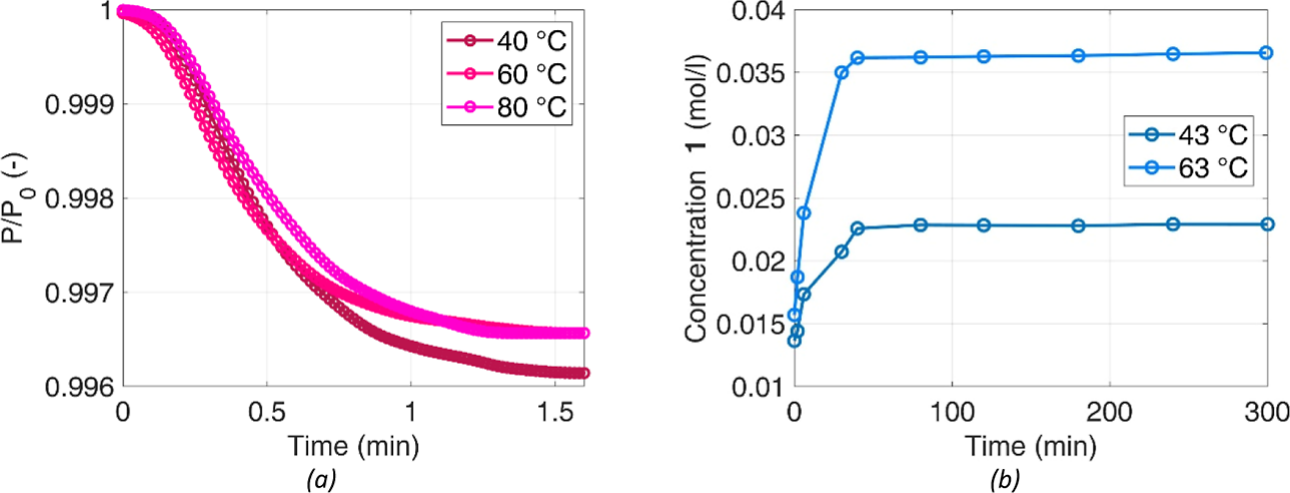
Solubility rate profiles at different temperatures: (a) normalized pressure of H_2_ and (b) concentration of **1**.

For H_2_, the pressure at the reactor headspace settled to a constant value after less than 1.7 min, at the lower temperature, meaning that saturation is reached very fast (Figure 4a). On the contrary, the time scale for **1** dissolution is larger; at 43 °C, the time to reach saturation is approximately 40 min, while it is 30 min for 63 °C (Figure 4b). It is experimentally confirmed that the rate of H_2_ transfer in the liquid is not a limiting factor for the reaction, evolving over a time frame of few minutes, while **1** dissolution rate is very likely to interfere with reaction kinetics.

### 4.3 Reference Test

**Figure 5:**
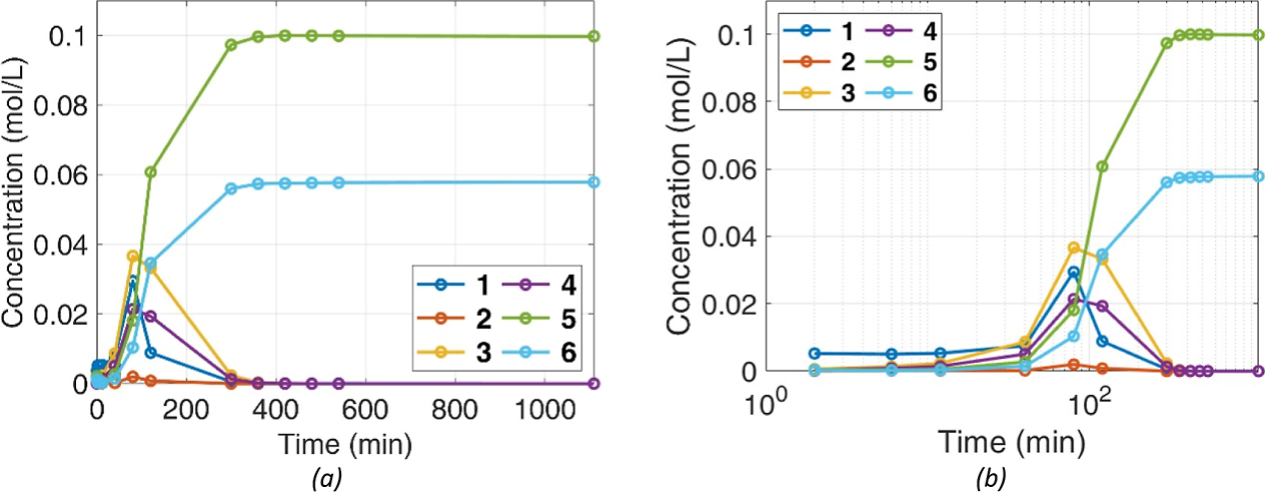
Species concentration in the reference test. (a) Linear and (b) logarithmic time scale.

The understanding of the process was initially developed at the laboratory scale, given the limited availability of costly reactants, as is frequently the case in pharmaceutical R&D. The results of a laboratory, batch experiment, at the reference condition and procedure described in the Experimental Section, are shown in Figure 5, as concentration profiles for all organic species in the liquid mixture. During the first 80 min of the process, the concentration of **1** increases, due to its dissolution, and in parallel all other species are generated thanks to the reactions with H_2_. This confirms that dissolution and reaction take place in parallel and compete; until 80 min, the dissolution of **1** is faster than its consumption, but that fast occurs almost sequentially. Should the reaction be much faster than dissolution, the concentration of **1** in the liquid would approach zero. Conversely, a very fast dissolution of **1** would saturate the solvent immediately and a constantly decaying concentration in the liquid is expected. Additionally, during the first 40 min, where both temperature and stirring rate are halved with respect to their maximum value, both generation of organic species and **1** dissolution are slower. At a lower stirring rate and temperature, the availability of **1** in the liquid bulk is reduced, and the reaction is not able to proceed at a significant pace.

The time required to reach 98% of **5** final concentration (τ_98_) is 310 min; the test is stopped when all intermediates’ concentrations are undetected and the ratio between **5** and **6** is in the expected range of 64/36.

**Figure 6:**
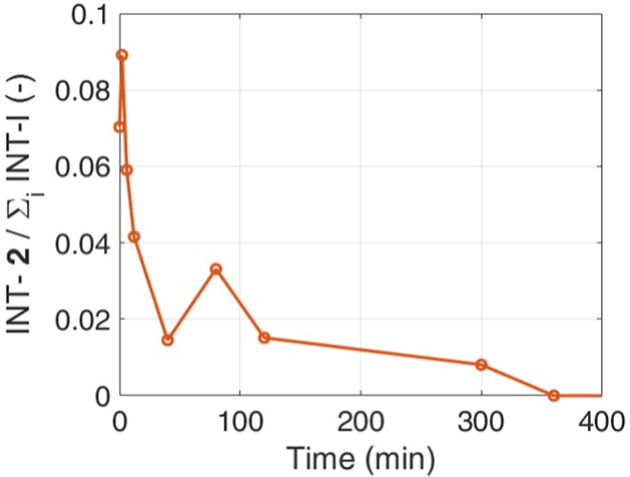
Ratio between concentration of intermediate **2** and the overall intermediate concentration (**2, 3,** and **4**).

Figure 5 also reveals the complex nature of the synthetic pathway: as soon as **1** dissolves, all intermediates and the products begin to appear in the mixture. The path through intermediates **3** and **4** is favored, as shown in Figure 6, that compares the concentration of intermediate **2** to the overall intermediate concentration (**2, 3,** and **4**). Initially, intermediate **2** constitutes approximately 10% of the total intermediates when it is scarcely present at the beginning of the reaction. However, this proportion rapidly decreases to less than 2% as the reaction progresses. At *t* = 80 minutes, the ratio between **2** and all intermediates increases. This is due to the difference in activation energies. The reaction leading to the formation of compound **2** exhibits a higher activation energy compared to those for compounds **3** and **4**. As the temperature increases from 40 to 80 °C, the production of compound **2** becomes more favorable. This results in an increased ratio of compound **2** to the total intermediates, explaining the observed behavior.

To further understand how process parameters impact the rates and selectivity and to what extent, a systematic investigation was planned.

### 4.4 Temperature Effect

**Figure 7:**
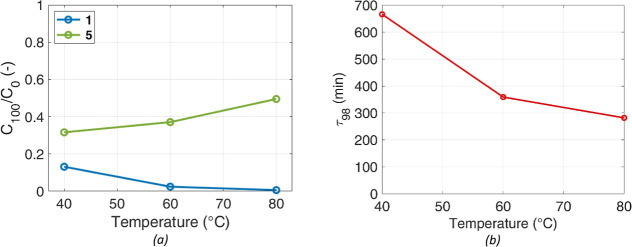
Temperature effect. (a) Concentration of **1** and **5** at *t* = 100 min, at different temperature, scaled to the initial theoretical concentration, *C*_0_. (b) Time required to reach 98% of **5** final concentration.

Tests were carried out varying the temperature in the range 40 to 80 °C. The temperature of the mixture was kept constant by the autoclave jacket. Since the beginning, stirring rate, catalyst loading, and pressure were kept constant at 600 rpm, 3.9% wt. (mass of catalyst/mass of solvents), and 8.5 bar, respectively. Figure 7a compares the concentrations of **1** and **5** after 100 min in these experiments, normalized by the initial theoretical concentration of **1,** assuming complete dissolution in the liquid phase. For clarity, only two of the six species are shown: the concentration for **1** describes the efficiency of solid/liquid mass transfer, as it is the only species involved in dissolution, and **5**’s profile is used to assess the success of the reaction. **6** mirrors **5**, at a smaller concentration.

An increase in temperature has a positive effect on both dissolution and chemical kinetics, as expected. At higher temperatures, **1** dissolution is faster, its saturation concentration increases, and all reaction rates increase. Accordingly, the higher the temperature, the faster the conversion of **1**. It is challenging to isolate the effects of dissolution and reaction. Indeed, the consumption of **1** on the catalytic surface decreases its concentration in the liquid, resulting in a driving force for dissolution which is much higher compared to the one for dissolution without reaction. **5** production rate is enhanced by an increase in temperature; τ_98_ values decrease exponentially from 40 to 80 °C (Figure 7b). The major differences are between experiment at 40 and 60 °C, as the reaction time is halved. However, keeping the temperature at 80 °C still makes the process 30 min faster than the reference one, while at 60 °C, the reaction takes 50 min more to complete.

**Figure 8:**
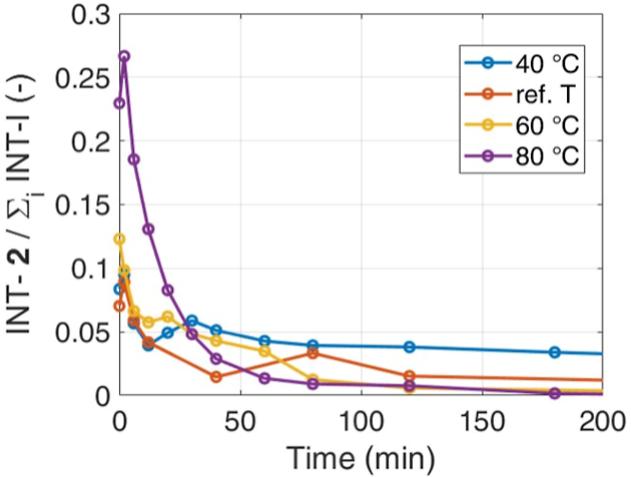
Effect of temperature on the ratio between concentration of **2** and all intermediates (**2, 3,** and **4**).

Figure 8 reveals that temperature favors the reaction path through the intermediate **2**, suggesting that the reaction rates that lead to **2** have a higher activation energy compared to those leading to intermediates **3** and **4**. This is an important finding, as it is known that the path through intermediate **2** leads to nonselective and incomplete reactions, so even though the increase in temperature is beneficial for reducing the process time, it is detrimental for the product purity. At 40 °C, the ratio remains higher even at extended reaction times. This behavior can be attributed to the reduced reaction rate at lower temperatures for the formation of products **5** and **6**.

### 4.5 Stirring Rate Effect

The impeller rotational speed, *N*, affects the degree of suspension of solid particles and the slip velocity (relative velocity between fluid and solid particles) and consequently the mass transfer coefficients. A common method to estimate the degree of particle suspension is the calculation of the ratio between the actual impeller rotation speed, *N*, and the one at just-complete suspension condition, *N*_S_, usually estimated by visual observation or with the Zwietering correlation
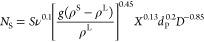
14

The solid/liquid mass transfer coefficient *k*_SL_ is related to the degree of particle suspension through correlations like^20^
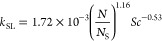
15Accordingly, increasing *N* will lead to a substantial improvement in mass transfer coefficients between the solid and liquid. Actually, there are two solid phases: the catalyst and the undissolved **1**. The loading of catalyst particles, *X*_cat_, is less than 5%, below the threshold of validity of the original Zwietering correlation. For low solid loadings, an exponent of 0.097 for *X*_cat_ can be replaced in eq 14.^21^ The predicted *N*_S_ for the catalyst is estimated to be 720 rpm on the laboratory scale, which is slightly higher than the value of 600 used in the standard procedure. However, it has been well established that achieving “complete” suspension requires considerably more power than achieving “almost complete” suspension (i.e., 98% of particles suspended), with the final few particles having negligible impact on the reaction.^22^ Visual observation (see Figure S5 in the Supporting Information) suggests that the catalyst is well dispersed in the solvent and very uniformly black colored. Also, the catalyst is made of fine particles (*d*_p,av_ = 94 μm) fully entrained in the liquid. For undissolved **1**, the Zwietering correlation predicts *N*_S_ = 1194 rpm. With *N* = 600 rpm used in the adopted procedures, the correlation predicts a degree of suspension *N/N*_S_ = 0.52, at the beginning of the process, when *X*_**1**_ is 11.7%. As the solid dissolves, the minimum stirring speed for just suspension lowers significantly since both particle diameter and solid loading decrease; then, keeping the same speed along the dissolution results in an increase of the suspension degree.

With this knowledge, two experiments at constant *N* were performed to assess its impact at 300 and 1000 rpm. The latter is a value much larger than that of the reference test and closer to the *N*_S_ suggested by the correlation. The temperature program, catalyst amount, and pressure were equal to the reference procedures.

**Figure 9:**
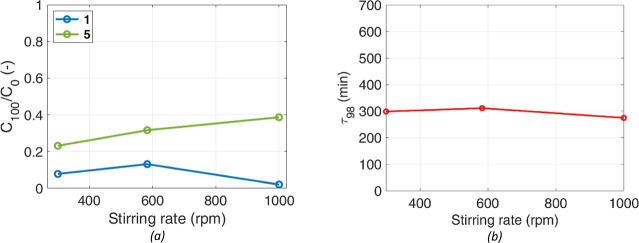
Stirring rate effect. (a) Concentration of **1** and **5** at *t* = 100 min, at different stirring rates, scaled to the initial theoretical concentration, *C*_0_, and (b) time required to reach 98% of **5** final concentration.

The reactant **1** profile in Figure 9a confirms that the stirring speed certainly affects dissolution and kinetics. As the stirring rate increases, the concentration of **1** at 100 min in the reference test is higher compared to that observed at 300 rpm. This is attributed to the increased dissolution rate, which makes more reagent available in the liquid phase; it is a consequence of the higher degree of solid suspension. Furthermore, a higher stirring speed determines an increase in slip velocity between the solid and the liquid, which reduces the diffusion film around the solid particle. This is further confirmed by the higher concentration of product **5**, measured with a larger stirring rate. On the other hand, at the highest stirring speed, **1** dissolves completely in a shorter time, but its concentration at *t* = 100 min is close to zero, as the reagent is more rapidly converted into intermediates and subsequently into final products; indeed, **5** concentration reaches the highest value at *N* = 1000 rpm. Such an unexpected impact of the stirring speed on the concentration of **1** in the liquid (increase and decrease) is just the result of the shift of the peaking profile shown in Figure 5 along the time, as the stirring is varied. Data points at earlier reaction times and throughout the process were collected for all these experiments, providing a strong basis for the interpretations discussed.^12^

Although an enhancement of solid/liquid mass transfer rate has a positive effect on dissolution, from the comparison of the τ_98_ trends, it is clear that temperature is far more important (Figures 7b vs 9b); **1** dissolution when temperature is lower is slow. A slightly positive influence of the stirring rate can be seen in the test with a higher stirring rate. Indeed, the **5** production rate is faster, but, finally, τ_98_ values do not differ significantly, with 35 min being the difference between the slower and the faster test, i.e., 10% of the total time. This confirms that the mass transfer from the liquid bulk to the catalyst surface is not a limiting factor for the process, as batch time is not significantly affected when the stirring speed is increased from 300 rpm, well below the predicted just-suspended condition for the catalyst, to 1000 rpm. Nevertheless, the process can still be considered mass transfer limited as the dissolution rate of reagent **1** is comparable to the reaction rate itself. Then, the stirring rate becomes almost irrelevant after the total dissolution of **1**, since H_2_ remains at saturation in the liquid and the kinetics controls the process. **1** dissolution completes when it reaches its maximum concentration in the solvent; afterward, **1** in the liquid is only consumed by the reaction. So, the stirring rate plays a major role just in the initial phases of the reaction, whose duration can be a significant part of the whole synthesis.

Interestingly, the stirring rate appears to affect also the selectivity. At higher *N*, the production of intermediate **2** is favored. At 1000 rpm, the initial ratio between **2** and total intermediate concentration is 2.6 times higher compared to the reference experiment (see Figure S6 in the Supporting Information). It can be a confirmation that the competition between the 2 synthetic pathways is ruled by the first reaction period, when dissolution is in progress.

### 4.6 Catalyst Amount Effect

**Figure 10:**
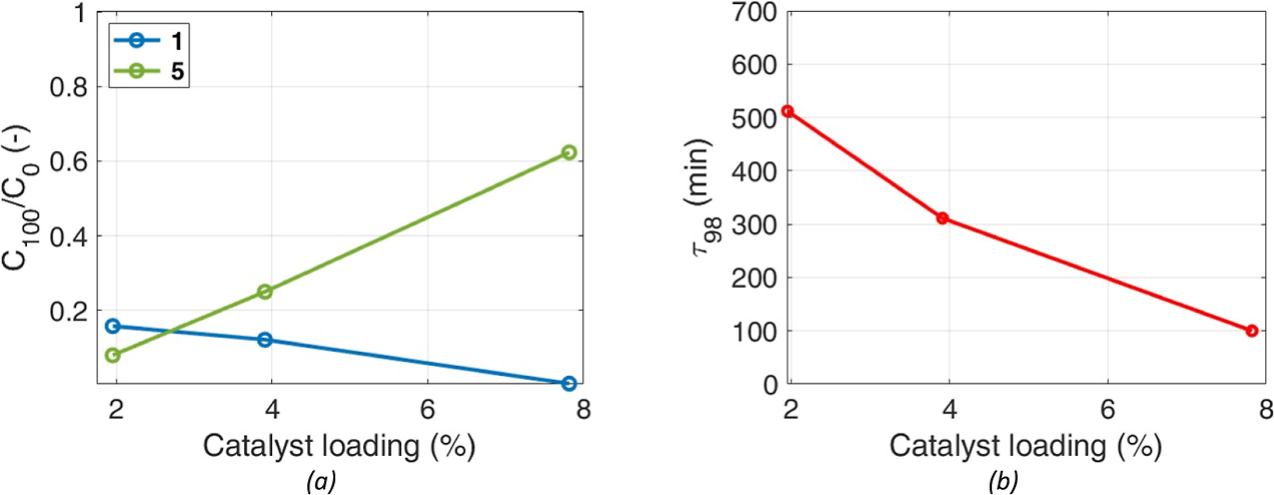
Amount of catalyst effect. (a) Concentration of **1** and **5** at *t* = 100 min, at different catalyst loadings, scaled to the initial theoretical concentration, *C*_0_, and (b) time required to reach 98% of **5** final concentration.

The impact of the catalyst amount was assessed doubling (7.80 wt %) and halving (1.95 wt %) the reference loading of Pd/C. All other parameters were kept equal to the reference test. Figure 10 shows the obtained results.

As expected, the reagent consumption is faster with more catalyst; **1** accumulates less in the liquid mixture (smaller peaks in its evolution), being rapidly turned into intermediates. Indirectly, a higher consumption rate on the catalyst surface also impacts the gradients of **1** in the liquid, which are the driving force for its dissolution. The combined effect of improved dissolution and reaction rates is apparent in the batch duration τ_98_ (Figure 10b and Table 1).

**Table 1:** τ_98_ and Apparent **5** Production Rate for Experiments at Different Catalyst Amounts

*X*_cat_ (%)	τ_98_ (min)	*r*_5_^exp^ × 10^–4^ (mol/min)	TOF × 10^–2^ (1/min)
1.95	510	0.83	4.90
3.90	311	1.58	4.70
7.80	97	3.91	5.80

Production of **5** clearly benefits from the increase in catalyst mass; all of the rates of production of intermediates and products are enhanced. Doubling the amount of the catalyst reduces the batch time by approximately 2/3, more than just halving. Indeed, the amount of the catalyst is expected to affect the rates of reaction, directly connected to reaction time only in simple chemistry and kinetics. The apparent rate of production of **5**, *r*_5_^exp^, was calculated as a time derivative of the **5** experimental concentrations, in the time interval after complete dissolution of **1**; values are reported in Table 1. *r*_5_^exp^ is confirmed to be linearly proportional to the mass of the catalyst charged; a nearly constant value of the turnover frequency (TOF) is obtained (last column in Table 1). Such a linear influence of the amount of the catalyst on the production rate explains two opposite cases, (i) a process controlled by the mass transfer rate from the bulk of the liquid to the catalyst surface or (ii) a chemically controlled surface reaction, with a pseudo first order, consistent with a large availability of H_2_ at the surface.^23^ Furthermore, the constant value of the TOF over the studied range of catalyst mass is also consistent with the use of an LHHW-type mechanism. While a variation in the catalyst amount would typically result in a decrease in the TOF if catalyst saturation is reached, the constancy of this value under the current operating conditions suggests that the process remains within the linear region of the reaction rate versus catalyst mass curve. In all cases, the process is linearly proportional to the solid–liquid interface area, which scales linearly with the number of catalyst particles present, *N*_P_, having the same particle diameter, or with the catalyst mass. This result implies that, in the case of kinetic control, the concentration of H_2_ at the catalytic surface is constant, and the reaction rates solely depend on the organic species concentration in the liquid. Such a result was also confirmed by a simpler model.^12^ Combining the effect of the catalyst amount with the one of temperature, it can be speculated that the rate of product formation from the intermediates is mainly controlled by kinetics, which is not surprising because these reactions develop mostly after total **1** dissolution, where a physical process was controlling.

As mentioned in Section 3.1, the presence of two distinct solid phases in the mixture may cause deviations from the ideal behavior of the conventional hydrogenation triphasic (G-L-catalyst) reactor. The Pd/C particles could influence the dissolution of the reagent, as frequent collisions between particles could improve the rate of reagent transfer from the solid to the liquid phase. The average distance between particles (Δ) was calculated for those of solid **1** and for the catalyst with eq 4. The results are summarized in Table 2, together with the total number of particles and the ratio between the average distance and particle diameter (Δ/*d*_p_).

**Table 2:** Average Distance between the Catalyst and **1** Particles at Varying Catalyst Loadings

*X*_cat_ (%)	*N*_p,cat_ × 10^5^ (−)^a^	Δ_cat_ (μm)	Δ_cat_/*d*_p,cat_ (−)	N_p,1_ × 10^5^ (−)^b^	Δ_1_ (μm)	Δ_1_/*d*_p,1_ (−)
1.95	36	317	3.38	6.1	453	1.54
3.90	72	232	2.47			
7.80	144	164	1.75			

a *d*_p,cat_ = 94 μm.

b *d*_p,**1**_ = 294 μm.

The values of Δ/*d*_p_, for the catalyst, at different loadings, suggest that the distance between particles is between approximately 2 to 3 diameters; it is reasonable then to assume that the dissolution of the reagent in the reaction mixture may be influenced by collisions with smaller catalyst particles. These collisions may break larger particles or conglomerates of **1**, thereby accelerating the transfer of the reagent to the liquid phase. In particular, at loadings of 3.90% and 7.80%, the average distance between each catalyst particle is even less than the initial average diameter of **1**.

For particles of **1** at the start of the process, Δ/*d*_p_ is 1.54. This is due to the relatively high initial value of *X*_1_ (11%) and the large size of such particles. This suggests that collisions between **1** particles of varying sizes could also influence its dissolution. This effect is more significant during the early stages of the process and diminishes as more of the solid phase dissolves. Given these results, it can be reasonable to incorporate in the developed model the correlation between the volumetric mass transfer coefficient for solid dissolution and the catalyst loading, eq 5, to account for these fluid dynamic and physical effects.

### 4.7 First-Principles Model

**Figure 11:**
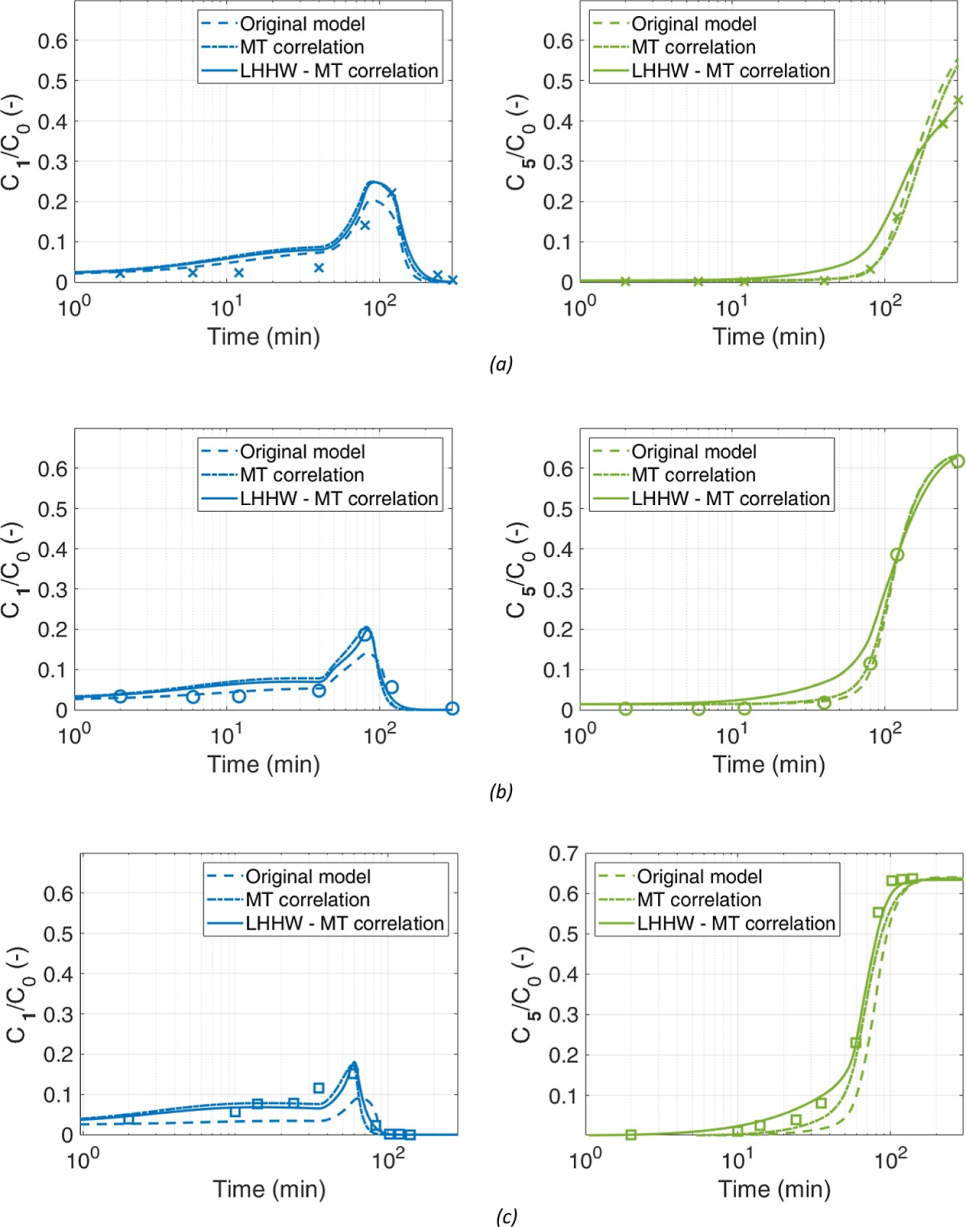
Different models’ predictions (lines) vs experimental data (symbols) for **1** and **5** scaled concentrations, after parameter optimization. (a) *X*_cat_ = 1.95%. (b) *X*_cat_ = 3.90%. (c) *X*_cat_ = 7.80%.

A mechanistic model to describe the four-phase hydrogenation reaction, accounting for limitations and distortions caused by the slow dissolution of a reactant, was previously developed.^12^ It was able to accurately predict the process, quantitatively accounting for the effects of temperature, stirring rate, reactor scale, and geometry. Its predictions in the tests with varying catalyst loadings presented above exhibited poor agreement with the measurements, and specifically the evolution of **1** in the solvent, while dissolution was in progress. This lack of a correct account of the role of the catalyst amount also affects the prediction of product evolution. According to the discussion in the previous section, the amount of the catalyst affects also the dissolution rate; the correlation, eq 5, between the mass-transfer coefficient for **1** in the liquid, at its solid interface, *k*_SL_a^SL^, and *X*_cat_ was incorporated in the model and calibrated with the experimental data. The results are summarized in Figure 11, where the improvement in the predictions of the model with the modified mass-transfer (MT) correlation is highlighted.

In addition, to further improve the accuracy of the chemistry, a microkinetic model accounting for the adsorption and desorption of species on the catalyst, leading to the rate expressions (8) and (9), was also implemented. The results of the different models’ predictions, at varying catalyst loading, for reagent **1** and product **5** are shown in Figure 11. Model predictions for the other product, **6**, are identical, as they are produced in a fixed ratio of 64/36. Note that a logarithmic time scale was used, to emphasize the beginning of the process, when dissolution is controlling the overall rate.

The suggested correlation for the mass transfer coefficient for dissolution allowed proper description of the positive effect of the amount of the catalyst on the evolution of **1** in the liquid, across all values of *X*_cat_. Accounting for an improvement of the mass transfer coefficient (*k*_SL_) due to a higher density of solid particles in the mixture, which increases the likelihood of particle collisions and conglomerate disaggregation, brings model predictions closer to the experimental data. Without accounting for the enhancement in dissolution, the model consistently underestimated the maximum concentration of reactant **1** in the solvent, particularly at the highest amount of catalyst loading; once **1** dissolves, it rapidly reacts to form the intermediates due to the increased kinetics resulting from the larger available catalytic area. Accordingly, the improvement in the prediction of the available reagent in the solvent is also reflected on the prediction of the products. A higher predicted concentration of the reagent in the liquid supports an increased reaction rate for intermediate formation and, consequentially, products.

Furthermore, the values are more consistent with the literature for hydrogenation reaction on Palladium (see Table S4 in the Supporting Information).^17,23−25^ This suggests that a more precise description of the critical dissolution process enables an improved identification of the intrinsic kinetics.

As expected, extending the reaction rate model with LHHW-type kinetics further improved the predictive capabilities across all values of *X*_cat_ (see Figure S7 in the Supporting Information). Improvements are particularly evident in the product quantification, especially at longer reaction times. However, the prediction of **1** did not improve significantly, further highlighting the prevailing importance of accurately describing its dissolution, i.e., the physical rather than the chemical process.

## 5 Conclusions

An experimental analysis for fed-batch catalytic hydrogenation for the production of Argatroban was performed. The process was revealed to be controlled by a slow dissolution of the starting reagent, that evolves in parallel to the reaction.

To identify and properly investigate the coupling of kinetics and mass transfer phenomena between different phases, a quantitative measurement of the actual reagent concentration in the liquid mixture is mandatory. The frequently used LCAP method was proved to dramatically mislead the identification of the actual process evolution and its controlling steps.

An extensive laboratory-scale campaign provided information about the effect of process parameters on the batch time and product purity. Increasing the reactor temperature enhanced both dissolution and kinetics to a different extent, resulting in significant reduction of process time; the variation from 40 to 80 °C reduced the batch time by 58%. Temperatures higher than 80 °C were detrimental to product purity, as they favor the formation of the undesired intermediate.

Stirring rate proved to be mostly relevant during the initial phases of the reaction, when reagent dissolution is a controlling step. A higher degree of particle suspension and an increase in slip velocity enhance mass transfer rates, thus reducing dissolution time and increasing reagent concentration in the liquid. A further 10% reduction of the process time can be achieved with higher agitation, but formation of undesired intermediates was observed.

Catalyst amount was highly influential on batch time reduction, as expected; the experiments allowed us to conclude that the process is first order in substrate concentration. While confirming that H_2_ was always at saturation in the solvent, a first-order influence of the reactant concentration was proved consistent with both mass transfer control at the beginning and a kinetic control later. The two regimes were singled out by the effect of varying the catalyst amount. Its impact on dissolution was based on the estimation of the average distance between particles of the two solid phases present in the reaction mixture; it can be concluded that collisions between reagent and catalyst particles could play a role in improving the dissolution of the reagent. An increase in catalyst loading enhances the likelihood of these collisions, thereby improving the mass transfer coefficient for dissolution.

A previously developed first-principles model that demonstrated predictive capabilities across a range of temperature, stirring rate, reactor scale, and geometry proved unable to account for the effect of the catalyst amount, assuming its impact was just on the kinetics. The model was improved to account for the role of catalyst particles in accelerating the reactant dissolution. A novel correlation between the solid–liquid mass transfer coefficient and the catalyst amount significantly improved the model’s accuracy in predicting reagent dissolution, at varying catalyst loading. This more accurate description of physical phenomena resulted also in a more precise prediction of products and a better identification of intrinsic reaction kinetics.

The extension to a microkinetic description of the reactions, accounting for adsorption and desorption, further improved model’s performance for intermediates and product profiles, but it did not substantially change the prediction of the dissolving reagent, further highlighting the critical importance of accurately describing the physical phenomena in such processes. The improved model can be utilized to guide and organize production campaigns involving multipurpose reactions. It also enables in-silico process optimization for pilot and production reactors, facilitating the identification of optimal operating conditions and the simplification of process workflows.

## Supporting Information Available

Experimental setup and conditions, additional research data, reaction model derivation, parameters, and comparison (PDF)

## Nomenclature

*A*^αL^: total interfacial area between phase α and liquid phases (m^2^)
*C*_*i*_^LC,int^: concentration of species “*i*″ at the catalyst–liquid interface (mol/m^3^)
*C*_*i*_: concentration of a single species in the liquid mixture (mol/L)
*D*: impeller diameter (m)
*d*_pα_: particle diameter of the solid in phase α (m)
*d*_pα,av_: average particle diameter of the solid phase α (m)
*K*_*i*_: adsorption equilibrium constants of species “*i*” (L/mol)
*k*_i_: kinetic constants
*k*_SL_: solid/liquid mass transfer coefficient (m/s)
*m*_p_: mass of the solid particle (g)
*m*_Pd/C_: catalyst mass (g)
*N*: impeller speed (rev/s)
molar flux of species “*i*″ from phase α to phase β (mol/m^2^ s)
*N*_*i*_^L^: number of moles of species “*i*” in the liquid phase (mol)
*n*_1,0_: initial number of moles of 1 (mol)
*N*_p_: number of solid particles (−)
*N*_S_: minimum impeller speed for complete suspension of the particles (rev/s)
*n*_tot,L_: total number of moles in the liquid phase (mol)
*Q*: heat of chemisorption (J/mol)
*R*_g_: universal gas constant (J/mol K)
*R*_*j*_: rate of reaction “*j*”, per unit surface (mol/m^2^ s)
*r*_*i*_: rate of species “*i*″ production, per unit surface (mol/m^2^ s)
*S*: Zwietering constant (−)
*Sc*: Schmidt number ν*/D*_*i*_ (−)
*T*: process temperature (°C)
*V*: reaction volume (m^3^)
*X*: weight fraction of the solid with respect to the liquid (%)

## Greek letters

α: generic phase name
β_*i*_: model parameter (−)
Δ_α_: average distance between solid particles of phase α (m)
δ_*ij*_: Kronecker’s delta, 1 for *i* = *j*, 0 for *i ≠ j*
ϵ_*r*_: relative error (−)
ν: kinematic viscosity (m/s^2^)
ν_*ij*_: stoichiometric coefficient of species “i″ in reaction “j″ (−)
ρ_L_: liquid-phase density (kg/m^3^)
ρ_S_: solid-phase density (kg/m^3^)
τ_98_: time to reach 98% of **5** final concentration (min)
*a*^αL^: specific area (= total/*V*^L^) between α and liquid phases (1/m)
