# Supplementary material for: Impact of Reactant Dissolution in the Kinetics of a Catalytic Hydrogenation for the Production of Argatroban
Source: Org Process Res Dev. 2025 Mar 12;29(3):735–47. doi: 10.1021/acs.oprd.4c00479 (PMC11934128; doi:10.1021/acs.oprd.4c00479)
Supplement: Supplementary file 1 — op4c00479_si_001.pdf [file op4c00479_si_001.pdf]

Electronic Supplementary Information

for

The impact of reactants dissolution in the kinetics of a catalytic hydrogenation for the production of Argatroban

Filippo Nanto*[a], Dario Ciato[b], Mariano Stivanello[b] and Paolo Canu[a]

[a] Industrial Engineering Department, University of Padova, via Marzolo 9,35131, Padova, Italy
[c] Lundbeck Pharmaceuticals Italy, Quarta Strada 2, Padua, 35129, Italy

*Corresponding authors
E-mail: filippo.nanto@phd.unipd.it

S1.1 Laboratory scale setup

The total experimental setup for the hydrogenation experimental campaign is shown in Figure S1.

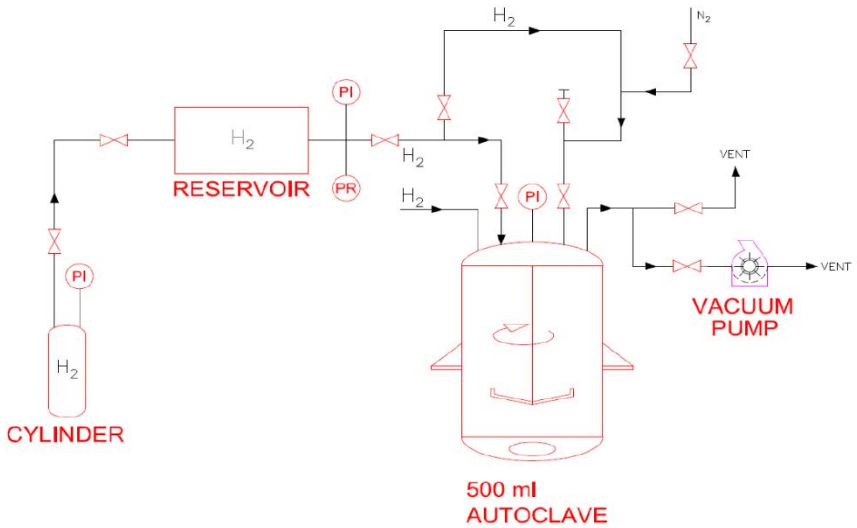

Figure S1 Experimental setup

Table S1 Laboratory reactor dimensions and ratios

Dimension or Ratio	
Tank diameter (T)	0.075 m
Tank cylinder height (H)	0.130 m
Stirrer diameter (D)	0.045 m
Impeller clearance (Cl)	0.035 m
Blade width (W)	0.009 m
H/T	1.73
D/T	0.60
W/D	0.20
Cl/D	0.78

The jacketed glass autoclave by *Buchi* has a full capacity of 800 ml and a nominal on of 500 ml, it was selected among the others available due to a geometrical similarity with the available pilot reactor. The autoclave is fully jacketed. RHODORSIL heat transfer oil is used both for heating and cooling operation. The source of the hydrogen for the laboratory scale experiments is a pressurized cylinder, acting as an intermediate reservoir, to avoid losing plenty of hydrogen in case of leakage.

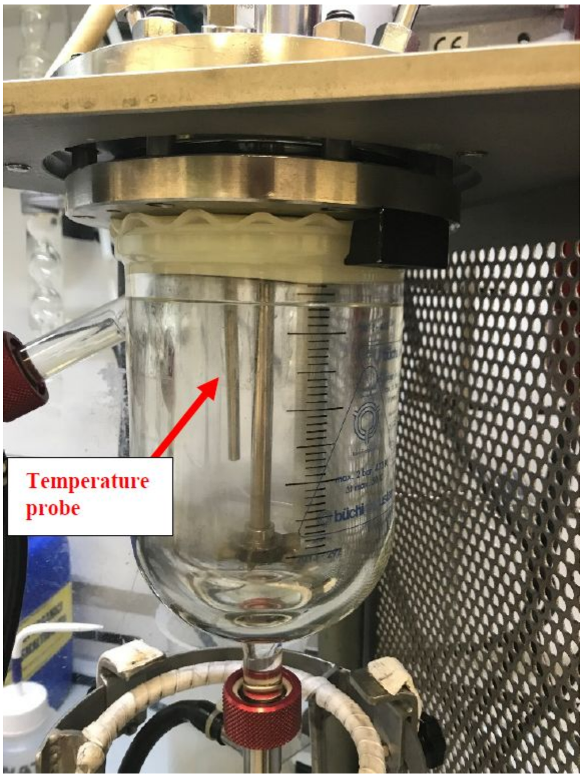

Figure S2 Laboratory autoclave equipped with temperature probe, that can also simulate baffle behavior

The impeller, Figure S3, is in stainless steel A304 with a resistance to corrosion and stress acceptable for this size.

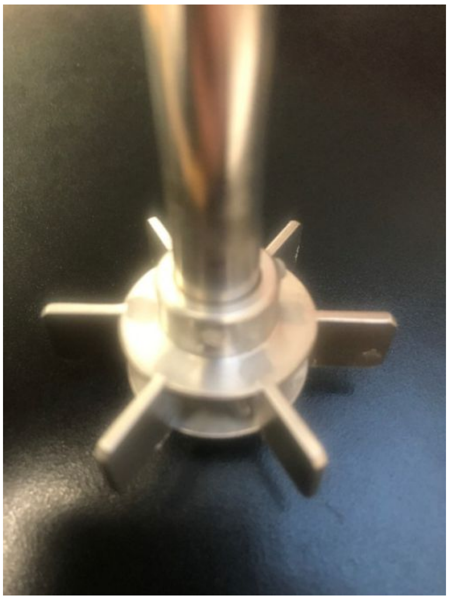
Figure S3 Self-inducing Rushton turbine, D = 0.045 m

S1.2 Experimental Conditions

The Table S2 summarizes the experimental conditions for the hydrogenation process.

Table S2 Parameters for hydrogenation process

Parameter	Notation	Value	Units
Nominal volume	V_N	0.500	mL
Methanol	V_{CH_3OH}	0.195	mL
Acetic acid	V_{CH_3COOH}	0.048	mL
Water	V_{H_2O}	0.004	mL
Mass of **1**	$m_{\mathbf{1},0}$	24.47	g
**1** mean diameter	$d_{p,\mathbf{1}}$	294	μm
Pd/C mass	$m_{Pd/C}$	7.16	g
Pd/C mean diameter	$d_{p,Pd/C}$	94	μm
Temperature	T_P	40-80	°C
Pressure	P_{H_2}	8.5	bar
Stirring rate	N	300-600	rpm

S1.3 HPLC method and analysis

The HPLC method was developed for internal use. The method was validated and is currently in use for the analysis of in-process control at the end of the hydrogenation process. All standards for all involved species were available: **1** and **5,6** are commercially available, while intermediates **2,3,4** were isolated from lab trials and purified via chromatography to obtain molecules with acceptable quality and in sufficient amount.

The HPLC method, using gradient elution and a hydrophobic column, is presented in Table S3 Description of HPLC method.

Table S3 Description of HPLC method

Element	Requested materials/actions
Mobile phase A	10 mM aqueous solution of ammonium acetate + 5 mM solution of sodium 1-heptansulphonate
Mobile phase B	Acetonitrile/Methanol (5:3)
Elution	Gradient
Column	*Inertsil* ODS-3 250 x 4.6 mm x 3µm
Detector	UV, 259 nm
Temperature	50 °C
Flow rate	0.6 mL/min
Injection volume	10 µL
Wash solvent	Methanol
Autosampler temperature	4 °C
Blank	4 mL methanol + 1 mL glacial acetic acid
Sample solution pretreatment	Suspension collected is filtered with 0.5 µm-cellulose
Sample solution	1 mL of elute diluted in 20 mL flask with methanol
Resolution isomers **5,6**	The resolution between peaks of **5** and **6** should be ≤ 1.2

Elution gradient:

Time (min)	Phase A	Phase B
0	60	40
20	60	40
35	50	50
50	20	80
60	20	80
60.1	60	40
72.1	60	40

S1.4 Standard test data

The concentration data on species evolution from HPLC was corrected based on the relative error between the total moles of all (reactive) species in the solvent (except H_2) and the initial moles of reactant initially loaded. In Figure S4 the original data set for the standard test is shown. As it can be seen the small oscillation in the products are adjusted by the correction.

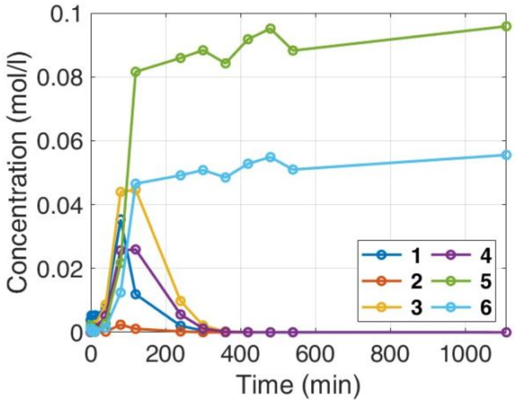

Figure S4 Raw data for the standard test at laboratory scale

S1.5 Pd/C suspension

It is not possible to use Zwietering correlation to calculate the minimum impeller speed to achieve complete suspension of particles, due to the low solid loading. It is not trivial also to recognize the quality of suspension by visual observation, due to the color of the suspension. However, generally the mixture appears to be uniformly black, indicating that the catalyst is well dispersed in the reaction volume, as it can be seen in Figure S5.

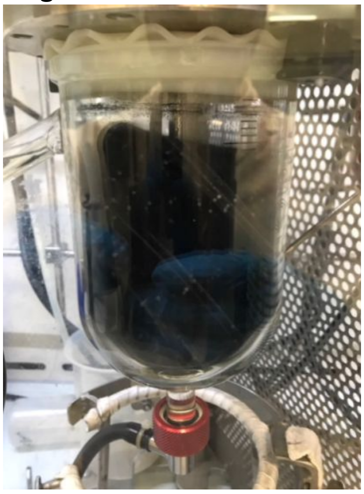

Figure S5 Reaction mixture with Pd/C suspended

S1.6 Stirring rate effect on intermediate formation

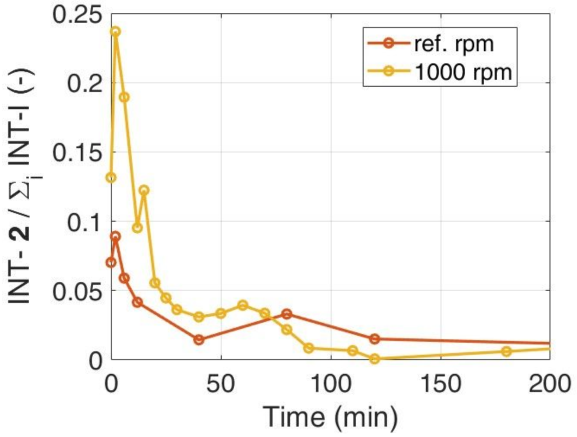

Figure S6 Effect of stirring rate on intermediates production

In the test at 1000 rpm, it can be noted an initial formation of **2** consistently higher in respect to the experiment at standard condition where initially the agitation rate is equal to 300 rpm (Figure S6). The profile at 1000 rpm goes to zero in less time because the process time is faster. The observed increase in ratio between the intermediates towards **2** at higher stirring rates likely results from the faster dissolution of reagent **1**, which accelerates the reaction before the temperature increases from 40 to 80 °C. This however leads also to a more rapid consumption of intermediates **3** and **4**, as their pathways to **5** and **6** are favored at lower temperatures, due to the lower activation energy, rather than an intrinsic change in selectivity.

S1.7 LHHW complete model

More complex and comprehensive formulations for the reaction rate expressions were developed for both the hydrogenation of the NO_2 group and the quinolinic ring. However, it was decided not to use these formulations in the model due to the high number of parameters required for fitting, which would likely result in good adherence to experimental data but also in strong correlations between the parameters.

The complete pathway for the hydrogenation of NO_2 follows the elementary steps shown in Scheme S1, where σ and π represent the active sites on the catalyst surface.

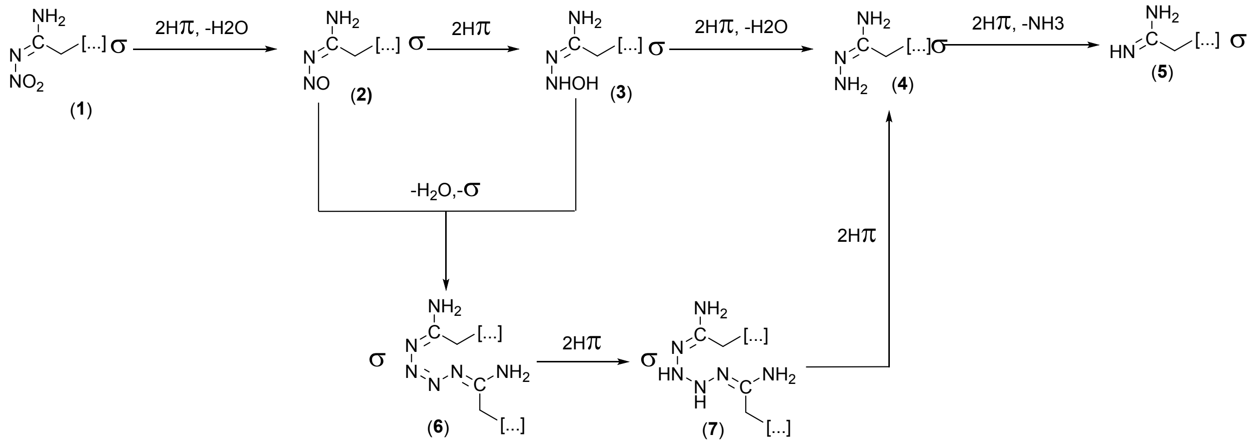

Scheme S1 Elementary steps in the hydrogenation of the NO2 group

The condensation pathway (through intermediate (**6**) and (**7**)) was not considered, given the absence of experimental evidence of the formation of such complexes. The series of elementary

steps can be summarized as followed, if non-competitive adsorption is assumed between hydrogen (π sites) and organic species (σ sites):

$$ads: (\mathbf{1}) + \sigma = (\mathbf{1})\sigma$$

$$ads: H_2 + 2\pi = 2H\pi$$

$$1\ (RDS): (\mathbf{1})\sigma + 2H\pi \rightarrow (\mathbf{2})\sigma + H_2O + 2\pi$$

$$2: (\mathbf{2})\sigma + 2H\pi = (\mathbf{3})\sigma + 2\pi$$

$$3: (\mathbf{3})\sigma + 2H\pi = (\mathbf{4})\sigma + H_2O + 2\pi$$

$$4: (\mathbf{4})\sigma + 2H\pi = (\mathbf{5})\sigma + NH_3 + 2\pi$$

$$des: (\mathbf{5})\sigma = (\mathbf{5}) + \sigma$$

In which the hydrogenation from (**1**) to (**2**) is considered to be the rate determining step (RDS). Under the assumption of partial equilibrium for adsorption/desorption processes:

$$C_{\mathbf{1}\sigma} = K_{1,ads} C_{\mathbf{1}} C_\sigma$$

$$C_{H\pi} = \sqrt{K_{H,ads} C_H} C_\pi$$

$$C_{\mathbf{5}\sigma} = K_{5,ads} C_5 C_\sigma$$

Where $K_{i,ads}$ are adsorption equilibrium constants. Assuming partial equilibrium also for all the reactions except for the rate determining step, the following expressions can be derived:

$$C_{\mathbf{2}\sigma} = \frac{K_2 C_{3\sigma} C_\pi^2}{C_{H\pi}^2}$$

$$C_{3\sigma} = \frac{K_3 C_{4\sigma} C_\pi^2 C_{H2O}}{C_{H\pi}^2}$$

$$C_{4\sigma} = \frac{K_4 C_{\mathbf{5}\sigma} C_\pi^2 C_{NH3}}{C_{H\pi}^2}$$

Where K_i are the equilibrium constants of the reactions. Combining this information, the expression of the adsorbed species in relation to the active sites concentration and the species in the liquid phase can be obtained:

$$C_{\mathbf{2}\sigma} = K' \frac{C_5 C_\sigma C_{H2O} C_{NH3}}{C_{H_2}^3}$$

$$C_{3\sigma} = K'' \frac{C_5 C_\sigma C_{H2O} C_{NH3}}{C_{H_2}^2}$$

$$C_{4\sigma} = K''' \frac{C_5 C_\sigma C_{H2O} C_{NH3}}{C_{H_2}}$$

Where K',K'',K'' are constants that are a combination of equilibrium and adsorption constants. The total concentration of σ and π sites is given by:

$$C_{\sigma,tot} = C_{\mathbf{1}\sigma} + C_{2\sigma} + C_{3\sigma} + C_{\mathbf{4}\sigma} + C_{\mathbf{5}\sigma} + C_{\sigma}$$

$$C_{\pi,tot} = C_{\boldsymbol{\pi}} + C_{H\pi}$$

From which an expression of C_{σ} and C_{π} can be isolated, given all the previous relations. The RDS reaction rate is expressed as:

$$R = k_1 C_{\mathbf{1}\sigma} C_{H\pi}^2$$

Combining equations and including $C_{\sigma,tot}$ and $C_{\pi,tot}$ inside the kinetic constant, since they are also assumed invariant, R can be expressed as:

$$R = \frac{k_1' K_{1,ads} K_H C_1 \, C_{H_2}^2}{\left(1 + K_{1,ads}C_1 + K_{5,ads}C_5 + K' \frac{C_5 C_{NH3} C_{H2O}}{C_{H_2}^3} + K'' \frac{C_5 C_{NH3} C_{H2O}}{C_{H_2}^2} + K''' \frac{C_5 \, C_{NH3}}{C_{H_2}}\right)\left(1 + \sqrt{K_H C_{H_2}}\right)^2}$$

Regarding the hydrogenation of the quinolinic ring, the complete pathway is shown in Scheme S2.

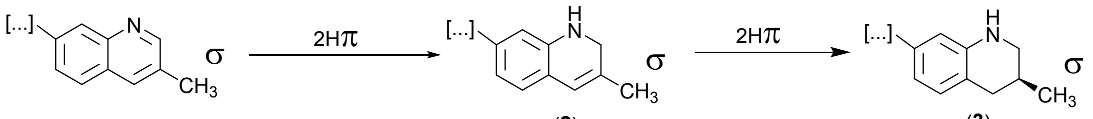

Scheme S2 Pathway for the hydrogenation of the quinolinic ring

So, the series of elementary steps can be summarized as followed, if non-competitive adsorption is assumed between hydrogen (π sites) and organic species (σ sites):

$$ads: (1) + \sigma = (1)\sigma$$
$$ads: H_2 + 2\pi = 2H\pi$$
$$1\ (RDS) : (1)\sigma + 2H\pi \rightarrow (2)\sigma + 2\pi$$
$$des: (2)\sigma = (2) + \sigma$$

$$ads: (2) + \sigma = (2)\sigma$$
$$2\ (RDS) : (2)\sigma + 2H\pi \rightarrow (3)\sigma + 2\pi$$
$$des: (3)\sigma = (3) + \sigma$$

Following the same assumptions and rationale used for the NO_2 reduction, but considering as RDS both the hydrogenation reactions, it is possible to obtain the expressions for the two hydrogenation reactions.

$$R_1 = \frac{k_1' \, K_{1,ads} \, K_H C_1 C_{H_2}}{(1 + \ K_{1,ads} \, C_1 + \ K_{2,ads} \, C_2 + \ K_{3,ads} \, C_3)\left(1 + \sqrt{K_H C_{H_2}}\right)^2}$$

$$R_2 = \frac{k_2' \, K_{2,ads} \, K_H C_2 C_{H_2}}{(1 + \ K_{1,ads} \, C_1 + \ K_{2,ads} \, C_2 + \ K_{3,ads} \, C_3)\left(1 + \sqrt{K_H C_{H_2}}\right)^2}$$

Which is in accordance with literature models for consecutive hydrogenations of double bonds [18]. However, species (**2**), which corresponds to the intermediate with only one hydrogenated double bond, was not detected in the liquid phase. Consequently, to mitigate the risk of overfitting and for this additional reason, a simplified version of the expression was selected for use in the model, that considers just the hydrogenation of the first double bond as the RDS. The series of elementary step was then assumed to be:

$$ads: (1) + \sigma = (1)\sigma$$
$$ads: H_2 + 2\pi = 2H\pi$$
$$1\ (RDS) : (1)\sigma + 2H\pi \rightarrow (2)\sigma + 2\pi$$
$$2 : (2)\sigma + 2H\pi = (3)\sigma + 2\pi$$
$$des: (3)\sigma = (3) + \sigma$$

In which the hydrogenation from (**1**) to (**2**) is considered to be the rate determining step (RDS). Under the assumption of partial equilibrium for adsorption/desorption processes:

$$C_{\mathbf{1}\sigma} = K_{1,ads} C_{\mathbf{1}} C_{\sigma}$$

$$C_{H\pi} = \sqrt{K_{H,ads} C_H} C_{\pi}$$

$$C_{\mathbf{3}\sigma} = K_{3,ads} C_{\mathbf{3}} C_{\sigma}$$

Where $K_{i,ads}$ are adsorption equilibrium constants. Assuming partial equilibrium also for the second reaction except for the rate determining step, the following expression can be derived:

$$C_{\mathbf{2}\sigma} = \frac{K_2 C_{3\sigma} C_{\pi}^2}{C_{H\pi}^2}$$

Where K_i are the equilibrium constants of the reactions. Combining this information, the expression of the adsorbed species in relation to the active sites' concentration and the species in the liquid phase can be obtained:

$$C_{\mathbf{2}\sigma} = \frac{K_2 K_{3,ads} C_3 C_{\sigma}}{K_{H,ads} C_{H_2}}$$

The total concentration of σ and π sites is given by:

$$C_{\sigma,tot} = C_{\mathbf{1}\sigma} + C_{2\sigma} + C_{3\sigma} + C_{\sigma}$$

$$C_{\pi,tot} = C_{\boldsymbol{\pi}} + C_{H\pi}$$

From which an expression of C_σ and C_π can be isolated, given all the previous relations:

$$C_{\sigma} = \frac{C_{\sigma_{tot}}}{K_{1,ads} C_1 + K_{3,ads} C_3 + \frac{K_2 K_{3,ads}}{K_{H,ads}} \frac{C_3}{C_{H_2}}}$$

$$C_{\pi} = \frac{C_{\pi_{tot}}}{1 + \sqrt{K_{H,ads} C_{H_2}}}$$

The RDS reaction rate is expressed as:

$$R = k_1 C_{\mathbf{1}\sigma} C_{H\pi}^2$$

Combining equations and including $C_{\sigma,tot}$ and $C_{\pi,tot}$ inside the kinetic constant, since they are also assumed invariant, R can be expressed as:

$$R = \frac{k_1 K_{1,ads} K_{H.ads} C_1 C_{H_2}}{\left(1 + K_{1,ads}C_1 + K_{3,ads}C_3 + \frac{K_2 K_{3,ads}}{K_{H,ads}} \frac{C_3}{C_{H_2}}\right)\left(1 + \sqrt{K_{H,ads}C_{H_2}}\right)^2}$$

Which is the simplified expression used in the model.

S1.8 Model parameters

All of the kinetic constants k_j follows the Arrhenius law, reformulated as:

$$k_j = exp\left[k_{0,j}" - \frac{Ea}{R_g}\left(\frac{1}{T} - \frac{1}{\bar{T}}\right)\right]$$

In this from the contribution of k_0'' and E_a are more balanced, reducing their correlation during fitting. $\bar{T}$ is the temperature in the middle of the investigated range, i.e., 60 °C.

Table S4 displays the kinetic parameters of the original model developed at constant catalyst concentration, and the ones found with additional measurements at different catalyst loading.

The latter are obtained with the extended model, with the mass transfer correction for the dissolution of the reagent, that accounts for the effect of collisions between particles. Incorporating the mass transfer correction allows the activation energy estimates of to be more consistent, among different reactions in the mechanism, and with values reported in literature for hydrogenation reactions catalyzed by Pd/C.

Table S4 Optimal Arrhenius parameters values found with the original model and the model parameters accounting for the mass transfer correction

	**Original model**		**Mass transfer correction**	
	k_0'' (1/min)	Ea (kJ/mol)	k_0'' (1/min)	Ea (kJ/mol)
k_1	-3.56	2.34	-3.64	15.88
k_2	-3.95	2.29	-4.14	15.66
k_3	-3.36	62.65	-6.34	24.04
k_4	-4.88	30.81	-4.91	28.80
k_5	-4.67	32.21	-4.74	21.65
k_6	-3.99	93.27	-4.15	36.20
k_7	-7.43	109.5	-8.34	32.10
β_1	-		8.53[a]	
β_2	-		3.02[a]	

[a] (-).

Table S5 displays the values of the kinetic parameters for the Langmuir-Hinshelwood-Hougen-Watson (LHHW) type model developed, together with parameters of the mass transfer correction expression.

Table S5 Model parameters values found with the LHHW model and the mass transfer correction

	LHHW model with mass transfer correction	
	k_0"	Ea (kJ/mol)
k_1	-3.03[a]	22.84
k_2	-3.35[a]	35.11
k_3	-3.31[a]	9.57
k_4	-4.37[a]	9.57
k_5	-5.95[a]	9.57
k_6	-0.06[a]	22.84
k_7	-1.18[a]	35.11
K_1	-0.04[b]	-2.67
K_2	-0.04[b]	-1.52
K_3	-3.59[b]	-177
K_4	-2.35[b]	-191
K_5	0.38[b]	-7.63
K_6	-0.03[b]	-9.28
K_H	-0.31[b]	-0.26
K_1	0.0001[c]	21.52
K_2	-0.45[c]	20.96

β_1	8.13[c]
β_2	2.89[c]

[a](mol/L*min). [b](L/mol). [c](-)

S1.9 Model comparison

Figure S7 displays the adherence to all the experimental data of the two different models, the global reactions model and Langmuir-Hinshelwood-Hougen-Watson (LHHW) type kinetic model. It is clear that the use of a more complex kinetic model improves model prediction, given also the increased number of parameters.

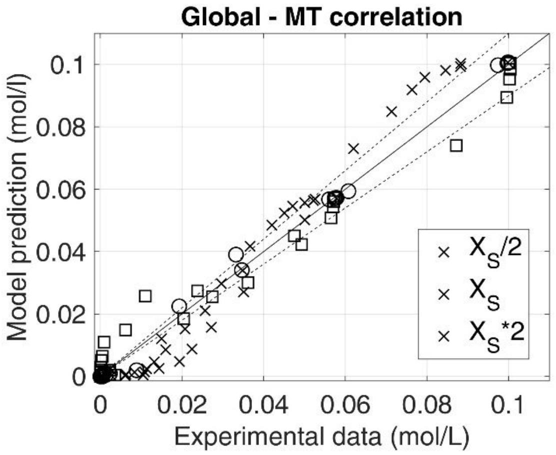

(a)

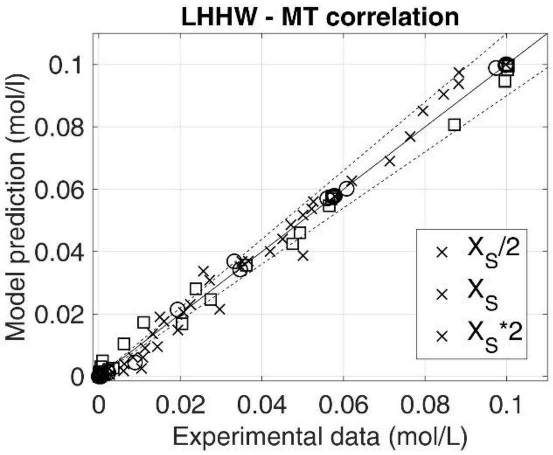

(b)

Figure S7 Parity plot: (a) Global reactions model with mass transfer correlation. (b) LHHW type model with mass transfer correlation
